# The Work of the R.A.M.C. in the War: The Regimental Medical Officer's Duties

**Published:** 1914-11-07

**Authors:** 


### THE WORK OF THE R.A.M.C. IN THE WAR.

### The Regimental Medical Officer's Duties.

BY A MEDICAL OFFICER AT THE FRONT.

In a previous letter (The Hospital, October 24, p. 85) a collection of war experiences were somewhat loosely strung together round the topic of field ambulance work. Important as the field ambulances are, they are not, of course, by any means the whole of the aid system for the sick and wounded in the field. The first link in the chain which connects the battlefield with the base consists of the regimental medical establishments. These include an officer of the Royal Army Medical Corps for each unit, and also a non-commissioned officer and half a dozen privates of the same corps for "water duties" and other hygienic affairs. These parties are "attached" to the particular battalion or other unit with whom they work. They draw rations, for instance, through the same source as their combatant colleagues, but they remain members of the Royal Army Medical Corps, and they continue to wear the "brassard" which protects them (or is supposed to protect them) under the Geneva Convention. They must not be confounded with the regimental stretcher-bearers, who are not members of the Royal Army Medical Corps, but are combatant privates trained in the elements of stretcher duties by the regimental medical officers. These stretcher-bearers number sixteen per battalion in the infantry, and for other units according to size. They act as a rule in peace time as bandsmen, and are trained also in the stretcher duties to which they revert in time of war. A certain amount of equipment is issued to each regiment, battery, or other unit, and a two-wheeled forage-cart is provided to carry it. This vehicle is not devised or suitable for the transport of wounded men, though a man wounded in the arm could easily be mounted upon it.

### The Paper Scheme in Practice.

It is the business of the regimental medical officer and his stretcher-bearers to gather up the wounded on the battlefield to such an extent as is practicable, to attend to their wounds and injuries as far as their resources allow, and to get them away from the immediate firing line if possible. The paper scheme of Royal Army Medical Corps work provides for the establishment of "regimental aid-posts"; that is to say, of sheltered places (buildings, sheds, valleys, or other places not under actual fire) to which the wounded may be conveyed. From these aid-posts the wounded are, theoretically, removed by the bearers sent up from the field ambulances to the advanced dressing stations, thence to be removed in the ambulance waggons further to the rear.

This regular sequence of removal is all very well on paper, but not so easy to maintain in practice. It overlooks, for one thing, the fact that the firing line is not in a fixed position, but is subject to rapid changes, forwards or backwards, or even both. The regimental aid-post of one moment may be occupied by the enemy if the forces retreat, or may be left far in the rear if they advance. Thus the regimental medical officer is constantly faced with the selection of new positions for his collecting post, as well as with the necessity of evacuating his wounded into the field ambulances as rapidly as possible. With the vast extensions of modern tactics, it is clearly impossible for the medical officer to be in more than a small fraction of the actual firing line at any one time, so that if he intends to succour the wounded actually where they fall he must travel long distances up and down a wide front, exposing himself necessarily to the enemy's fire in so doing. Nor is it desirable, from the strictly military point of view, that he should do this.

### Where the Wounded are Safe.

The wounded are far safer lying where they fall—provided someone will apply the first field-dressing which every soldier carries—than being taken away in daylight while the action is still proceeding. To attempt to carry wounded men, or to let the slightly wounded walk to the aid-posts during the heat of an engagement, merely exposes them to grave risk of additional wounds, and inevitably means losses among the medical *personnel*. This lesson has been driven home since the present campaign began by the high proportion of casualties among the regimental medical officers and stretcher-bearers, more especially the former. The Germans seem to have appreciated this much sooner than we did, for they have persistently refused to allow their medical officers and bearers access to the battlefield while the contest still raged. Presumably, their medical service has, in consequence, not to lament the loss of anything like the number of medical officers (proportionately) that we have. Experience, however, teaches; and our generals and senior medical officers are beginning to realise that in this matter considerations of sentiment must be subordinated to business. The regimental medical officer who travels up and down the firing line, or the trenches, at imminent risk of his life, may earn respect for his bravery, but none for his common sense. Combatant officers were far too much inclined at first to encourage this sort of bubble reputation-seeking among their medical officers, but they are now beginning to see that it does not pay. Another reason for avoiding this method of employing the regimental medical *personnel*, so expensive of trained men whom it is difficult to replace, is that in the nature of things only first-aid can be attempted under such conditions. For the majority of wounds the simple application of the emergency field-dressing is quite sufficient; and this can be done by any intelligent comrade, and does not demand the presence of a medical officer.

The proper position of the regimental medical officer, then, is the regimental aid-post; and this should be selected by him, having in mind the features of the tactical situation of the moment. In some cases it may be as near as one or two hundred yards to the firing line, in others it may need to be six or even eight hundred yards further back; as a general rough outline, perhaps three hundred yards in enclosed country and five hundred in open may be suggested as average distances.

As has already been said, the aid-post may need to be changed, forwards or backwards, during the course of an engagement. As to the necessity for this the medical officer has no sufficient means of judging; his time will be amply taken up by the care of the wounded. He should therefore be duly informed by the officer commanding the unit of any advance or retirement, and must of course be prepared to move his whole equipment at the shortest notice. One of his difficulties is that in the heat of battle the combatant commanders have a way of forgetting all about him, and of omitting to notify him of their intended movements. He may thus find his aid-post suddenly tumbling about his ears from shell fire, or himself and his bearers faced with a sudden retirement under heavy rifle fire, leaving the wounded to be captured. Or, in the other event, he may find himself a mile or two behind the fray, and exposed to reprimand or imputations of slackness.

### The Temptation of the V.C.

The point is that *it is not the business of the regimental medical officer* to go right up to the firing line: he should await the wounded in some safer place further back. If this entails that wounded men have to lie out on the field for hours at a time, that is unfortunate for them, but one of the consequences of war. It is better that a man should thus lie until darkness enables his removal to be conducted with safety than that he and a stretcher party and a medical officer should lose their lives through a misguided gallantry on the part of the latter. To lay down this principle is easier than to get it universally enforced. The temptation to run the gauntlet in the old-fashioned way, in the hope of winning a V.C. or some lesser decoration, is still very great, and will be so until general officers and commanding officers comprehend the situation and discourage their medical officers from unnecessary risks in attending the wounded under fire. Nor must it be suggested that medal-hunting is the inspiration of these exploits. There are many dashing officers in the Royal Army Medical Corps to whom such ventures appeal as "sporting propositions," if one may put it so. Such bold spirits must learn that their lives and their trained skill are too valuable to the Army and the country to be wasted, and that they must restrain their inclination to get right up to the firing line to pick up wounded.

Another important part of the regimental medical officer's duties lies in his relations with the nearest field ambulance, upon which he relies to remove the wounded at the earliest opportunity from the aid-post. He should be apprised, through the deputy assistant-director of medical services, the brigade-major, and the adjutant of the combatant unit to which he is attached, of the position of the nearest dressing station opened by a field ambulance (or detachment of one); and should then send written messages to the officer in charge there stating his own position, the number of wounded collected, how many of them can sit up, and similar points.

### A Delicate Matter.

Then, again, when there is no actual fighting going on, the regimental medical officer has plenty of important duties. It is his business to see the sick of his unit, to treat such of them as he can, and to forward the rest to a field ambulance for evacuation to a hospital. It has happened in consequence of the excessive mortality among regimental medical officers as already explained, that a great number of regiments are now being officered by specially enlisted members of the Royal Army Medical Corps. These gentlemen are often young and comparatively recently graduated, and few of them have any experience of Army conditions. Hence they are fair game for the scrimshanker and the malingerer, who "go sick" in large numbers and are frequently referred by the medical officer to the field ambulance when there is not the least necessity for it. The officer commanding the field ambulance is then confronted with a difficulty. He knows that the men should not have been sent to him at all, and that if he sends them on to the base hospitals he is helping to weaken the firing line as well as exposing himself to rebuke from senior officers at the base; but if he sends them straight back to the regiment he is administering a direct snub to a young man who has acted in good faith but has been imposed upon. Still, this latter alternative is that which is forced upon him by the exigencies of the service. Sometimes, no doubt, there is legitimate room for difference of opinion as to whether a man is or is not fit for duty; but in such a case the rule is to give him the benefit of the doubt by treating him as a case of unfitness and transferring him as soon as possible to the base. If he quickly recovers, he is sent up with the next reinforcing draft; if he gets worse, he is in the best conditions for treatment and recovery. It will be seen how manifold and important are the duties of the regimental medical officers, so many of whom have laid down their lives during the short time that the campaign has so far lasted.

